# Crosstalk between Microtubule Stabilizing Agents and Prostate Cancer

**DOI:** 10.3390/cancers15133308

**Published:** 2023-06-23

**Authors:** Qiao-Hong Chen

**Affiliations:** Department of Chemistry and Biochemistry, California State University, Fresno, CA 93740, USA; qchen@csufresno.edu; Tel.: +1-559-278-2394

**Keywords:** prostate cancer, microtubule stabilizing agent, androgen receptor

## Abstract

**Simple Summary:**

Prostate cancer is one of topmost health concerns, and metastatic castration-resistant prostate cancer is the fatal form. Two taxanes, docetaxel and cabazitaxel, are the only two FDA-approved chemotherapeutics that can provide survival benefits to patients with the lethal version of prostate cancer. Taxanes, alongside numerous other naturally occurring products, can promote the assembly and stability of microtubules to halt cell division and promote various cancer cell deaths. Additionally, this group of compounds, named microtubule stabilizing agents, can impede the androgen–androgen receptor complex from moving into the cell nucleus, conquering androgen receptor-containing prostate cancer cell proliferation and metastasis. This review aims to overview the preclinical and clinical studies, clinical uses, and the mechanisms of action of microtubule-stabilizing agents as anti-prostate cancer agents.

**Abstract:**

A variety of microtubule-stabilizing cytotoxic agents (MSA) with diverse chemical scaffolds have been discovered from marine sponges, microorganisms, and plants. Two MSAs, docetaxel and cabazitaxel, are the exclusive chemotherapeutics that convey a survival benefit in patients with castration-resistant prostate cancer (CRPC). Additional MSAs have been investigated for their potential in treating prostate cancer in both clinical and preclinical settings. Independent of promoting mitotic arrest, MSAs can suppress the nuclear accumulation of androgen receptor (AR), which is the driving force for prostate cancer cell growth and progression. The alternative mechanism not only helps to better understand the clinical efficacy of docetaxel and cabazitaxel for AR-driven CRPC but also provides an avenue to seek better treatments for various forms of prostate cancer. The dual mechanisms of action enable MSAs to suppress AR-null prostate cancer cell proliferation by cell mitosis pathway and to interfere with the AR signaling pathway in AR positive cells. MSA chemotherapeutics, being administered alone or in combination with other therapeutics, may serve as the optimal therapeutic option for patients with either castration-sensitive or castration-resistant prostate cancer. This review provides an overview of the anti-prostate cancer profiles (including preclinical and clinical studies, and clinical use) of diverse MSAs, as well as the mechanism of action.

## 1. Prostate Cancer

Prostate cancer remains one of top health concerns due to its highest and still rising incidence, as well as the fact that it is the second greatest number of cancer-related deaths in American men. In 2023, about 29% of all new cancer cases among the U.S. men were estimated to be prostate cancer, while over 34,700 prostate cancer deaths were projected to occur in the United States [1]. The androgen receptor (AR)-regulated transcriptional pathway provides the main impetus to prostate cancer cell growth and metathesis [2]. Due to the fact that the binding of androgen to AR initiates the transcriptional pathway, androgen deprivation therapy (ADT) has been a mainstay of treatments for prostate cancer since 1941. Unfortunately, the original response to ADT barely lasted for about 18 to 24 months [3]. The castration-resistant prostate cancer (CRPC) persists to progress even under extremely low levels of androgen in serum, and the metastatic CRPC contributes to the lion’s share of prostate cancer deaths in the United States. Docetaxel, a simesynthetic derivative of paclitaxel, has been approved by the U.S. Food and Drug Administration (FDA) in 2004 because it is the first chemotherapy that can offer a survival advantage to patients with metastatic CRPC [4]. Since then, the FDA has approved cabazitaxel, another paclitaxel derivative, alongside several therapeutics, as illustrated in Table 1, for treatments for, or diagnostics of, different forms of prostate cancer. These marketed therapeutics can be grouped into taxane-based chemotherapeutics: AR-targeted hormonal therapy, immunotherapy, and radiotherapy. It is worth noting that, so far, docetaxel and cabazitaxel are the only two chemotherapeutics that can significantly prolong overall survival time for patients with CRPC. Initiated by ADT, hormonal therapeutics have consistently remained the cornerstone of treatment for patients with CRPC since 1941. This notion is substantiated by the recent approval of five hormonal therapies (Table 1) by the U.S. FDA for the treatment of prostate cancer. Additionally, preclinical studies have demonstrated the promising therapeutic efficacy of MAPK signaling pathway inhibitors against enzalutamide-resistant prostate cancer [5,6]. Immune checkpoint inhibitors, in combination with a diverse array of immunomodulating agents, are currently being investigated in numerous clinical trials for their potential clinical use for patients with prostate cancer [7].

## 2. Microtubule Stabilizing Agents

**Figure 1:**
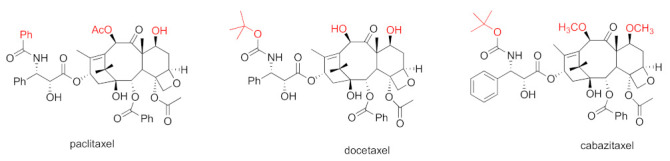
Chemical structures for paclitaxel, docetaxel, and cabazitaxel.

Taxane-based chemotherapeutics are defined as naturally occurring paclitaxel and its derivatives (Figure 1), which stop cell division and cause cell apoptosis via promoting microtubule assembly and stability [8]. Microtubules are tube-shaped dynamic protein polymers composed of α-tubulin and β-tubulin heterodimers. As main components of the cytoskeleton, microtubules are believed to be one of the vital druggable targets for cancers due to their critical role during the process of cell division and mitosis [9]. The taxanes are the pioneering and well-known class of microtubule stabilizing agents (MSAs) with three taxanes (paclitaxel, docetaxel, and cabazitaxel) (Figure 1) in the market as blockbuster anticancer drugs [10]. One main driving force for the success of taxane-based chemotherapeutics is the seminal discovery from Dr. Susan Horwitz’s lab; paclitaxel can accelerate microtubule assembly in vitro and stabilize microtubules in animal models which was, then, an inaugurated and unique mechanism of action for anticancer drugs [11,12]. The successful launch of paclitaxel in clinical use has spurred tremendous interest in seeking MSAs for treatment of various cancers employing a mechanism-based drug discovery strategy. Numerous MSAs with diverse chemical scaffolds and distinct binding sites/modes have been discovered from marine sponges, microorganisms, and plants [13]. The taxanes have been successfully used to treat different solid tumors for over 30 years [14]. Specifically, paclitaxel has been approved for the treatment of breast, ovarian, and lung cancer, together with Kaposi’s sarcoma. Nab-paclitaxel/abraxane has been used to treat breast, pancreatic, and lung cancer. Docetaxel has been used as the therapeutics for patients with head and neck, breast, lung, prostate, or stomach cancer. Cabazitaxel has been used for prostate cancer [14]. Additionally, ixabepilone (the semi-synthetic derivative of epothilone B) has also been approved for clinical use for patients with breast cancer [15].

Independent of blocking cell mitosis, obstructing intramolecular trafficking on microtubules has later been established as an alternative crucial mechanism of action for MSAs [16,17,18,19]. As the mere chemotherapy class that can provide survival benefit to patients with mCRPC, two MSAs docetaxel and cabazitaxel are the current first-line and second-line chemotherapeutics for mCRPC [4,20,21]. Docetaxel and cabazitaxel are semi-synthetic derivatives of naturally occurring paclitaxel (Figure 1). It has been evidenced that MSA paclitaxel interferes with AR trafficking, leading to the impairment of AR transcriptional activity by blocking nuclear importation of AR as a downstream of microtubule stabilization [22]. The newly added in mechanism not only enhances our understanding of the clinical effectiveness of docetaxel and cabazitaxel in treating AR-driven CRPC, but also opens up possibilities for exploring improved treatments for various forms of prostate cancer. The multifaceted mechanisms enable MSAs to not only suppress AR-negative prostate cancer cell proliferation via the cell mitosis pathway, but also to interfere with the AR signaling pathway in AR-positive cells [22]. MSAs may be an optimal therapeutic option for both castration-sensitive and castration-resistant prostate cancer. This review provides an overview of the anti-prostate cancer profiles (including preclinical and clinical studies, and clinical use) of MSAs, together with the mechanism of action.

## 3. Preclinical Studies of MSAs as Anti-Prostate Cancer Agents

Even though a plethora of MSAs with distinct chemical structures have been identified and their anticancer properties have been extensively explored in the preclinical settings, only a small portion of the MSAs have been investigated in prostate cancer cell models (Table 2), and prostate cancer xenografts in animal models.

### 3.1. Paclitaxel and Analogs (Taxanes)

Paclitaxel (**1**) was a naturally occurring diterpenoid compound isolated by Dr. Wall and Dr. Wani from the stem bark of Taxus brevifolia, commonly known as Pacific yew [27]. In vitro cytotoxic potency against leukemia cell lines (P-388 and P-1534) has been confirmed prior to the structural characterization by X-ray structure analysis in 1971. Paclitaxel was eventually able to bypass various barriers and progressed to the clinical development because of (i) its superb in vivo antitumor efficacy against colon and breast tumor xenografts; and (ii) the unique mechanism of action, at that time as it was identified, as the first microtubule stabilizing agent by Dr. Susan Horwitz [10]. Paclitaxel, alone or in combination with other drugs, has so far been approved by the U.S. FDA for the treatment of AIDS-related Kaposi sarcoma, breast cancer, non-small cell lung cancer, and ovarian cancer (https://www.cancer.gov/about-cancer/treatment/drugs/paclitaxel, accessed on 14 April 2023). The in vitro studies in 1992 revealed that paclitaxel at 1 nM can boost the capability of estramustine in suppressing androgen-independent prostate cancer cell mitosis [28]. The promising results motivated the further clinical studies of paclitaxel/docetaxel alone or in combination with estramustine (or other agents) for the potential treatment of CRPC [29,30]. Albumin-bound paclitaxel was developed to keep away from the toxicities associated with Cremophor/ethanol in Cremophor-based paclitaxel. Using the equivalent dose, the albumin-bound paclitaxel treatment led to more thorough regressions, delayed recurrence, prolonged doubling time, and a higher survival than the Cremophor-based paclitacel treament in five different xenografts, including prostate cancer xenograft [31].

Docetaxel, a semisynthetic derivative of paclitaxel, is two-fold more potent than paclitaxel in promoting the microtubule assembly with a broader anti-tumor spectrum [32]. Docetaxel possesses IC_50_ values ranging from 5 to 43 nM against several murine and human cancer cell lines [33]. Intravenous administration of docetaxel led to the complete shrinkage of eleven different kinds of xenograf tumors [33]. Docetaxel demonstrated the significant anti-tumor efficacy in a HID28 CRPC tumor xenograft model [34].

To overcome drug-resistant issues faced by paclitaxel and docetaxel, searching for the second-generation taxanes with improved medicinal properties has been pursued. Towards this end, the 7,10-O-dimethyl derivative of docetaxel, named cabazitaxel (Jevtana) (Figure 1), was chosen for further clinical studies from around 450 taxanes according to its superior capability in stabilizing microtubules, suppressing docetaxel-resistant cancer cell proliferation, and shrinking docetoxel-resistant xenograft tumors [35]. Cabazitaxel exhibited a greater potency than docetaxel in suppressing CL1 CRPC cell proliferation [36]. Cabazitaxel was revealed to possess an in vivo anti-tumor efficacy towards a broad spectrum of tumor models, including a DU145 prostate xenograft tumor model and a HID28 CRPC tumor xenograft. Importantly, cabazitaxel exhibits a greater in vivo anti-tumor efficacy than docetaxel in the CRPC xenograft model [34]. The greater anti-tumor efficacy of cabazitaxel, in both docetaxel-resistant and enzalutamide-resistant metastatic CRPC xenograft models, is correlated to its higher intratumoral concentration [37]. The preclinical safety profiles for cabazitaxel are similar to those for paclitaxel and docetaxel.

### 3.2. Epothilones

**Figure 2:**
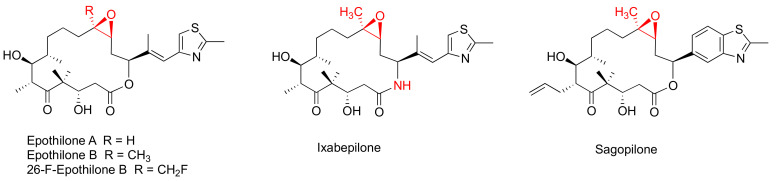
Chemical structures for representative epothilones.

Epothilones (Figure 2) are a group of 16-membered macrolide MSAs originally isolated from the myxobaterium Sorangium cellulosum [38,39]. Epothilones exhibited nanomolar antiproliferative potency in the NCI 60-cell line screening, including PC-3 and DU145 prostate cancer cell lines [38]. The structurally unrelated epothilones bind to the taxane pocket of β-tubulin using their side chains to structure an M-loop into a short helix [40,41]. Epothilone A, epothilone B, and 26-fluoroepothilone B (Figure 2) demonstrated low IC_50_ values of 0.5–4.0 nM against a panel of prostate cancer cell lines (PC3, LNCaP, MDAPCa 2a, and MDA PCa 2b) (Table 2). Further in vivo investigation on 26-fluoroepothine B against MDA PCa 2b and PC3 xenografts in athymic nude mice indicated that this epothilone B derivative possessed a superior antitumor efficacy to that of paclitaxe at equivalent doses [24]. Epothilone B, also known as patupilone or EPO906, at 4–5 mg/kg, effectively inhibited PC-3M and DU145 xenograt growth in athmic mice [42].

The most clinically advanced derivative of epothilones is ixabepilone (aza-epothilone B, BMS-247550), which is a semi-synthetic aza-derivative of epothilone B [43]. Not only does ixabepilone possess a greater anti-tumor efficacy than paclitaxel does towards paclitaxel-sensitive tumors, but it has superior activity against paclitaxel-resistant cancer xenografts [44]. Additionally, ixabepilone was revealed to have a broad-spectrum anti-tumor efficacy towards thirty-three cancer xenografts, including four prostate cancer xenografts [45].

### 3.3. Taccalonolides

**Figure 3:**
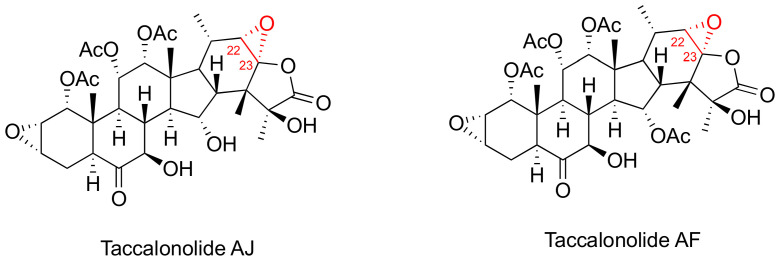
Chemical structures for representative Taccalonolides.

Taccalonolides (Figure 3) are a class of MSAs featuring a pertacylic steroid scaffold that was isolated from plants [46,47]. Certain taccalonolides are effective towards the drug-sensitive and multidrug-resistant cancer cells through arresting the G2-M cell cycle, and inducing BCl-2 phosphorylation and cell apoptosis. The C22-C23 epoxide moiety of taccalonolide AJ covalently binds to the carboxy group of amino acid D226 of β-tubulin. This changes the M-loop conformation which results in the assembly of tubulins into microtubules [48]. Extensive investigation on their in vitro and in vivo potency towards various cancer cells and xenografts have been performed [47]. For example, taccalonolide demonstrated a strong antitumor potency and efficacy in MDA-MB-231 breast cancer xenografts [46]. However, no preclinical studies has been reported so far for the taccalonolides in any prostate cancer cell and tumor models.

### 3.4. FR182877 (Cyclostreptin)

**Figure 4:**
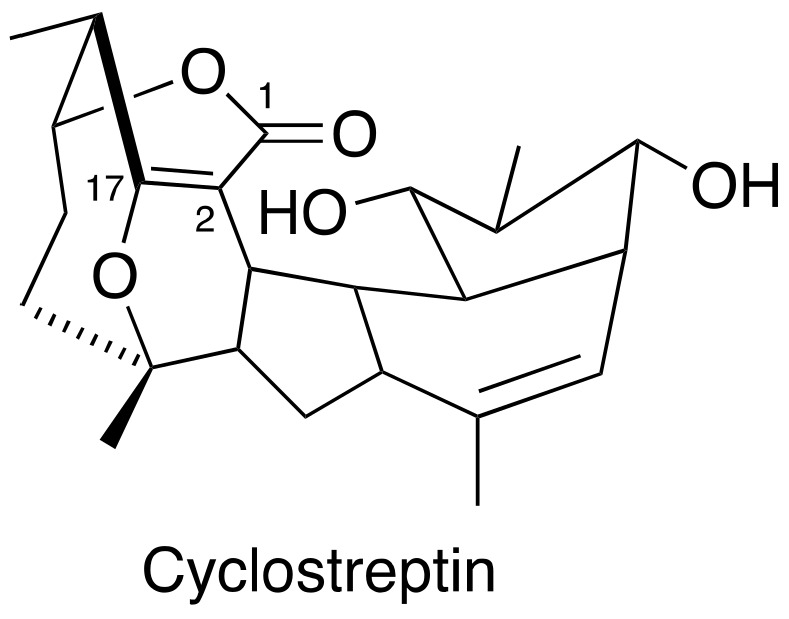
Chemical structure for cyclostreptin.

Cyclostreptin (Figure 4) was isolated from Streptomyces sp 9885, representing the first MSA covalently bound to microtubules. Even though cyclostreptin only moderately promotes tubulin assembly, it can actively bind to microtubules to block other MSAs from binding to the same microtubules. Cyclostreptin showed a promising antiproliferative potency in various cancer cells and antitumor efficacy against P388 and colon xenografts in mice models [49,50]. So far, no investigation was initiated in prostate cancer cells and xenografts.

### 3.5. Protopine

**Figure 5:**
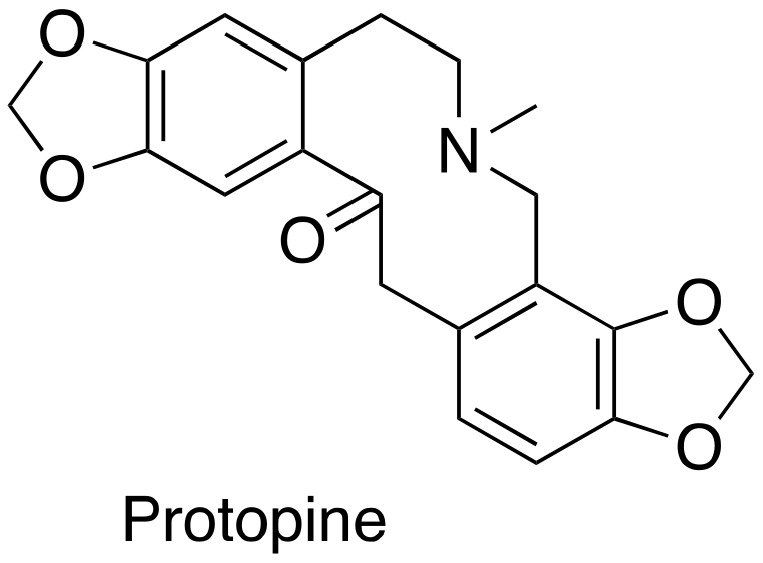
Chemical structure for protopine.

Protopine (Figure 5), an isoquinoline alkaloid, was established as a MSA with a simple chemical structure. Protopine can suppress DU145 and PC-3 cancer cell proliferation according to the SRB (sulforhodamine B) bioassay, which is associated with its capability of promoting mitotic arrest and prostate cancer cell apoptosis [25].

### 3.6. Other Representative MSAs

**Figure 6:**
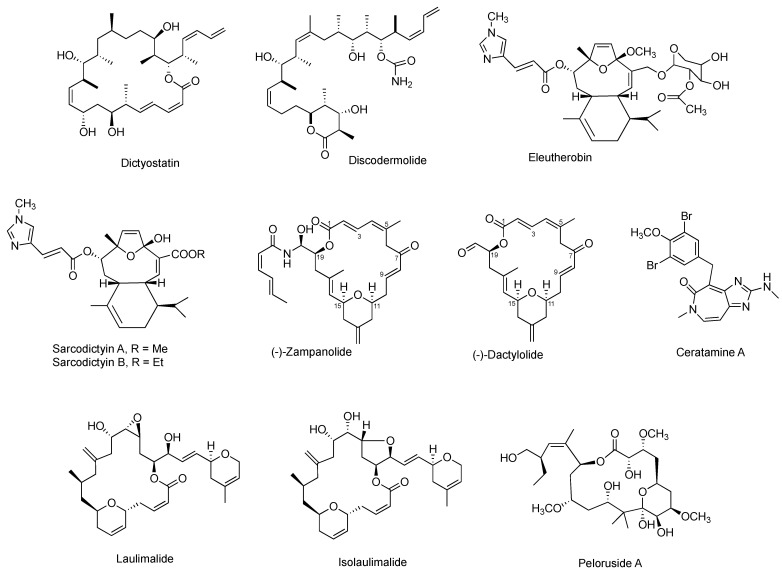
Chemical structures for other representative MSAs.

Plenty of other naturally occurring MSAs with very diverse chemical structures have been isolated from various natural sources. As illustrated in Figure 6, most of them originally isolated from marine sources with macrolides as the prevalent group. The majority of them can suppress cell proliferation towards at least one cancer cell line at low nanomolar concentrations. Some of their anti-proliferative potency has been supported by the promising anti-tumor efficacy in animal models. However, merely a few of them have been exploited for their in vitro antiproliferation in anti-prostate cancer cell models, and none of them has been assessed for their in vivo anti-tumor efficacy on prostate cancer xenografts (Table 3). The scarcity of the natural products is the main barrier preventing these MSAs from more preclinical and clinical studies.

Among them, pelorusdie, laulimalide, and isolaulimalide have a distinct binding site on β-tubulin, compared with other MSAs [51]. Instead of binding to the taxane luminal site of the microtubule, these three MSAs bind to the external surface of the microtubules [52]. Zampanolide and dactylolide bind to β-tubulin through a covalent bond between its C-9 carbon and N227 of β-tubulin [41].

**Table 3:** Reported Data of other representative MSAs as anti-prostate cancer agents.

Compound	Origin	Chemical Scaffold	In Vitro Evaluation	In Vivo Evaluation	Tubulin Binding Mode	Refs.
Dictyostatin	Marine sponge	Macrolide polyketide	N/A	N/A	reversible	[53,54]
Discodermolide	Marine sponge	Polyhedroxylated lactone	N/A	N/A	reversible	[55]
Eleutherobin	Marine coral	Diterpene glycoside	PC-3 DU145	N/A	reversible	[56]
Sarcodictyins	Marine coral	Diterpene	N/A	N/A	reversible	[57]
Zampanolide	Marine sponge	macrolide	PC-3	N/A	irreversible	[26]
Dactyolide	Marine sponge	macrolide	PC-3	N/A	irreversible	[26]
Laulimalide	Marine sponge	macrolide	N/A	N/A	reversible	[58]
Isolaulimalide	Marine sponge	macrolide	N/A	N/A	reversible	[58]
Peloruside	Marine sponge	macrolide	N/A	N/A	reversible	[59]
Ceratamines	Marine sponge	Heterocyclic alkaloid	N/A	N/A	reversible	[60]
Rhazinilam	plant	Biphenyl lactam	N/A	N/A	reversible	[61]

## 4. Clinical Studies and Use of MSAs for Patients with Prostate Cancer

### 4.1. Paclitaxel

On the grounds of its promising efficacy in patients with platinum-refractory ovarian cancer, paclitaxel alone was moved forward to the first clinical trial (Phase II) by the Eastern Cooperative Oncology Group in twenty-three patients with bi-dimensionally measurable CRPC [62]. The treatment regimen in this study is no more than six cycles, with each 21-day cycle consisting of a 24 h infusion at either 135 mg/m^2^ or 170 mg/m^2^, depending on the pre-treatment history. The clinical trial merely resulted in minor efficacy, as only one out of twenty-three patients responded to the treatment. The following clinical trial of a more intense paclitaxel treatment, in eighteen patients with progressive metastatic CRPC, suggested weekly one-hour paclitaxel at 150 mg/m^2^ showed a significant treatment efficacy [63]. In this trial, 50% of patients with a measurable disease achieved a major response; 39% of patients had a 50% or greater reduced-serum PSA level. The different efficacies between these two small clinical trials implies that a greater dose intensity and overall serum concentration plays a critical role for paclitaxel to treat CRPC.

In contrast, resonating with the preclinical profiles, the Phase I and Phase II clinical studies of paclitaxel/estramustine combination, for the potential treatment of metastatic CRPC, demonstrated more attracting results [29,30], which inspired more clinical studies aiming to validate the efficacy of paclitaxel, in combination with estramustine, for metastatic CRPC [64,65]. The patient numbers for these four clinical trials range from 21 to 63. Among them, 27% to 58% of the patients experienced a decline of 50% or greater in the serum PSA, and the median overall survival is from 16 to 19 months. However, a high rate of toxicity was observed with 37.5% of patients discontinuing the treatment within 4 weeks in the Phase I trial conducted by Fox Chase Cancer center and Indiana University [64]. There were 465 clinical trials on paclitaxel, as a single agent or in combination with other anticancer drugs for prostate cancer, according to the search results from ClinicalTrials.com.

### 4.2. Docetaxel

The initial Phase II clinical studies of docetaxel aimed to evaluate its therapeutic efficacy for patients with various solid tumors. Docetaxel was administered through a one-hour in vitro infusion at a recommended dose of 100 mg/m^2^, once every three weeks in this clinical evaluation. Docetaxel was concluded to be effective in treating breast, non-small-cell, ovarian, head and neck, gastric, melanoma, and soft tissue tumors [32]. The clinical use of docetaxel for the treatment of advanced breast cancer was approved in 1996 by the FDA. Two independent Phase II clinical trials (75 mg/m^2^) using docetaxel as a monotherapy for CRPC were initiated in 1999 [66,67]. Significant serum PSA response (defined as 50% reduction in serum PSA) rates (38% and 46%), disease response rates, and tumor regression were observed in both clinical trials.

Vinca-alkaloid vinblastine and estramustine are the two microtubule-destabilizing compounds that originally motivated scientists to delve into the potential of MSAs treating CRPC [68]. Considering vinblastine and estramustine bind to different sites of microtubules, three clinical trials were designed to evaluate the therapeutic effect of the combined vinblastine and estramustine on treating prostate cancer [69,70,71]. The combined treatment did not cause significant improvement in overall survival [72]. However, the significantly higher PSA response rate, longer time to cancer progression, and greater antitumor efficacy were observed from the combined treatment of vinblastine and estramustine, compared with vinblastine alone. These encouraging clinical data initiated the clinical studies of the combination of estramustine with docetaxel for CRPC.

Several clinical trials have been conducted for the combination therapy of paclitaxel for patients with CRPC [68,73,74,75]. The new clinical use of docetaxel plus prednisone, as the standard first-line treatment for patients with metastatic CRPC, was approved by the FDA in 2004 based on the survival benefit evidenced by two, critical Phase III clinical trials: Southwest Oncology Group 99-16 and TAX 327 [4,21]. In the TAX 327 clinical study, conducted from March 2000 through June 2002, 1006 patients with metastatic CRPC were randomly assigned into three groups. In addition to receiving 5 mg of prednisone twice daily, the first group administered mitoxantrone at 12 mg/m^2^ every three weeks as a treatment control; the second group administered docetaxel at 75 mg/m^2^ every three weeks as a treatment group; the third group administered docetaxel at 30 mg/m^2^ weekly for five of each six-week cycles. The treatment group with docetaxel every three weeks possessed the longest survival time (18.9 months), while the mitoxantrone control group had the shortest survival time (16.5 months). It was concluded that the combination of docetaxel/prednisone apparently improved survival, compared with that of mitoxantrone/prednisone in this Phase III study [21]. In the other Phase III clinical study, conducted by the Southwest Oncology Group, 770 men with CRPC were randomly assigned to the control group (mitoxantrone/prednisone) and treatment group (estramustine/docetaxel/dexamethasone) [4]. The findings from this clinical study suggest that an approximate two-month survival was achieved for the treatment group, compared with the control group. However, the combined docetaxel/estramustine increased severe side effects as evidenced by the increased mortality caused by the treatment or treatment interruption due to the adverse events.

Considering that the advert events caused by the docetaxel/prednisone could add in an extra burden to the men with deadly and aggressive CRPC, numerous clinical studies were conducted to test whether docetaxel is effective to treat patients with hormone-sensitive prostate cancer (HSPC) or even in an earlier non-metastatic stage [76]. It has now been revealed that the concomitant treatment with ADT plus docetaxel (named as chemohormonal therapy) resulted in a 13.6-month longer median overall survival, 8.5-month median delayed time to cancer progression, and 10.9% higher rate of patients with a less than 0.2 ng/mL PSA level at 12 months, than that with ADT alone [77]. The observed survival benefit is unprecedented in the field of prostate cancer, leading to 94 fewer deaths per 1000 men [78]. The 20.4-month prolonged overall survival was achieved by ADT plus docetaxel, compared with ADT alone, in patients with high-volume HSPC in the CHAARTED trial, with a more mature follow-up [79].

### 4.3. Cabazitaxel

In clinical studies, cabazitaxel in combination with prednisone, was used to treat patients with metastatic CRPC and with previous docetaxel treatment. The clinical observations of cabazitaxel resonated well with its preclinical profiles. According to the results obtained from the FIRSTANA clinical trial, treating CRPC patients with cabazitaxel or docetaxel resulted in a similar overall survival. The FDA approved cabazitaxel, in combination with prednisone, in June 2010, as a new therapeutic option for CRPC patients who have previously received docetaxel treatment [80,81]. This FDA approval was mainly based on the 2.4 months of overall survival benefit caused by the combined treatment of cabazitaxel and prednisone in the randomized Phase III TROPIC clinical trial [20]. Neutropenia and diarrhea are the main side effects of cabazitaxel treatment. The Phase II TAXYNERGY clinical trial, in men with chemotherapy naïve metastatic CRPC, concluded that the early switch between docetaxel and cabazitaxel served as a good strategy to improve PSA response rates [82], supporting the notion that different taxanes possess varying resistant mechanisms [83,84,85,86]. Another question was raised regarding the potential favorable or detrimental effects of long-term use of palliative prednisone. The favorable effects are attributed to the suppression of adrenal androgen and cytokine activity. However, the detrimental effects arise from the occurrence of adverse events, activation of AR variants, and the impact on cabazitaxel clearance through the induction of CYP3A4 [87]. This question promoted further investigation into the role of prednisone in cabazitaxel treatment, which led to a conclusion that excluding daily prednisone from the treatment regimen had no negative impact on both overall survival and safety profiles [87].

Cabazitaxel treatment for 255 patients with metastasis CRPC who had previously been treated with docetaxel, and the alternative abiraterone or enzalutamide, led to a 4.3-month prolonged imaging-based progression free survival and 22.2% increased PSA response rates, compared with continued abiraterone or enzalutamide treatment [88].

### 4.4. Ixabepilone, Epothilone B, and Sagopilone

Ixabepilone has entered Phase I clinical studies for patients with melanoma, ovarian cancer, non-small-cell lung cancer, and breast cancer, resulting in apparent antitumor responses and a recommended dosing schedule for Phase II studies [43]. Ixabepilone was assessed for its efficacy in patients with paclitaxel-resistant CRPC because it can suppress paclitaxel-resistant cancer cell proliferation [89]. During the period of December 2001 and October 2003, 92 patients with chemotherapy-naïve metastatic CRPC were assigned into two groups: ixabepilone treatment group and ixabepilone/estramustine treatment group [90]. The results from this multi-institutional Phase II clinical study, verified the clinical antitumor activity of ixabepilone, alone or in combination with estramustine, in patients with metastatic CRPC. Recently, ixabepilone (BMS-247550) was evaluated by the Southwest Oncology Group in 42 patients with chemotherapy-naïve metastatic CRPC [45]. It was concluded from this Phase II trial that ixabepilone treatment resulted in the 33% PSA response rate (14 out of 42 patients), 6-month estimated median progression-free survival, and 18-month median survival. The clinical studies of ixabepilone have been systematically summarized by Dorff et al. [91]. Additionally, the other two epothilones, epothilone B and sagopilone, were also revealed to have excellent efficacy in Phase II clinical trials in patients with CRPC [92,93,94,95].

## 5. The Mechanisms of Action of MSAs as Anti-Prostate Cancer Agents

### 5.1. Stop Cell Mitosis

Regarding the mechanism of action lying behind the clinical efficacy of MSAs in patients with CRPC, it is initially believed that the clinical efficacy of MSAs originated from their antimitotic activity [96]. As the essential components of the mitotic spindle, the dynamics of microtubules are indispensable for daughter chromosome segregation in all eukaryotes. MSAs interrupt the dynamics through binding to β-tubulin, and promoting the assembly and stability of microtubules, resulting in a cell-cycle arrest, mitosis inhibition, and eventual cell apoptosis [9].

### 5.2. Block AR Trafficking

Independent of suppressing cell mitosis, the clinical efficacy of MSAs is also linked to the AR trafficking dependent on microtubule [22]. Docetaxel was unexpectedly found to downregulate the expression of AR and PSA in AR-positive prostate cancer cells (LNCaP, CWR22Rv1, and MDA-PCa-2b) [97]. The down-regulation of PSA by docetaxel aligns with the appreciably higher PSA response rates observed in the clinical studies of MSAs, as mentioned in Section 4. The in vitro down-regulation of AR by docetaxel ties the clinical efficacy of MSAs in CRPC with the AR signal pathway. Another independent study reported that AR activity in 22RV1 prostate cancer cells can be suppressed by paclitaxel, as evidenced by down-regulated PSA and NKx3.1, as well as the inhibition of AR luciferase reported genes [98]. The inhibition of AR activity by paclitaxel has been revealed to be mediated by an AR-suppressive nuclear transcription factor named FOXO1 [98]. Further in vitro and clinical studies found that docetaxel can impair AR nuclear trafficking because the docetaxel-treated tumors had decreased nuclear AR accumulation, accompanied with an increased cytoplasmic AR concentration [99]. Paclitaxel was then found to suppress the subsequent AR transcriptional activity, as characterized by the ARE (androgen response element) luciferase reporter vector in LNCaP prostate cancer cells.

AR nuclear translocation was not blocked by MSAs in cells with acquired β-tubulin mutations that hinder microtubules from being promoted to stabilization by MSAs, further corroborating that microtubules play a crucial role in AR trafficking [100]. A further study indicated that AR trafficking is mediated by dynein: a motor protein located at the minus-end of microtubules. After being activated by a ligand, the AR is loaded to the dynein and then is shuttled on microtubules from the cytoplasm towards the nucleus, where the AR binds to DNA and eventually leads to prostate cancer cell proliferation and metastasis. MSAs chemotherapies act in part by preventing the AR from moving into the nucleus through binding to the β-tubulin and stabilizing microtubules.

The interaction between AR and α-tubulin was detected in two AR-positive prostate cancer cell lines (LNCaP and CWR22) by yellow immunofluorescence staining [99]. The DNA binding domain and hinge region on the AR were identified as the microtubule-dynein binding domains by comparing the docetaxel sensitivity in vitro and in vivo, and the nuclear accumulation and transcriptional activity of ARv7 and ARv567 [101]. ARv7, lacking the hinge region, did not co-precipitate with dynein motor protein nor with microtubules, resulting in a resistance to docetaxel in vitro and in vivo. Docetaxel treatment cannot affect the nuclear ARv7 accumulation and ARv7 transcriptional activity. In contrast, docetaxel treatment is very effective in the LuCaP86.2 tumor xenografts that express ARv567, and docetaxel can increase the nuclear ARv567 accumulation and transcriptional activity. Arguably, it was reported that both ARv7 and ARv567^es^ diminished the responses to taxane treatment in LNCaP prostate cancer cells [102].

### 5.3. Induce Bcl-2 Phosphorylation

It was reported that MSAs (e.g., paclitaxel) treatment can induce phosphorylation of Bcl-2 in prostate cancer cells and other cancer cells that express Bcl-2, leading to cell apoptosis [103,104]. MSAs interfere with the integrity of microtubules, which promotes Bcl-2 phosphorylation. Docetaxel is 100-fold more potent than paclitaxel in inducing Bcl-2 phosphorylation and cell apoptosis.

## 6. Conclusions and Future Perspectives

So far, MSAs are the only chemotherapy class with significant survival benefits for patients with CRPC. This triggered a lot of effort on exploring their intrinsic mechanisms of action as anti-prostate cancer drugs (agents). It is now clear that the clinical efficacy of MSAs for patients with CRPC is not only associated with their antimitotic activity but also linked to the AR trafficking, which is dependent on microtubules. Inducing phosphorylation of Bcl-2 in prostate cancer cells was the other reported mechanism of action for the MSAs to suppress prostate cancer cell proliferation. The multiple mechanisms not only help to better map the clinical benefits of MSAs for AR-driven CRPC but also to set up a solid foundation to search for better treatments for various forms of prostate cancer. The multiple mechanisms of action enable MSAs to suppress AR null prostate cancer cell proliferation by the cell mitosis pathway and to interfere with the AR signaling pathway in AR positive cells. MSA chemotherapeutics, being administered alone or in combination with other therapeutics, may serve as the optimal therapeutic option for patients with either castration-sensitive or castration-resistant prostate cancer.

Additionally, further delving into microtubule-dynein-AR signal pathway will help to identify the molecular basis behind the taxane resistance in the patients with CRPC. The established understanding about the AR trafficking, using microtubules as shuttles, not only support the synergistic clinical benefits brought by the combination therapies of androgen antagonists and MSAs for CRPC, but also provide a clearer guidance for designing better combined therapies for CRPC based on the inhibition of two distinct but synergistic pathways in the AR signaling axis. Given the motor protein dynein serves as a needed part in transporting the AR via the microtubule-dynein-AR axis, it emerges critical to understand why dynein motor protein can specifically recognize the AR. This information will set up a solid foundation for rationally designing dynein protein inhibitors that would be used alone or in combination with taxane chemotherapies for the potential treatment of CRPC. Also, a decreased percent of AR nuclear localization (%ARNL) and an increased microtubule bundling can serve as early biomarkers for the therapeutic efficacy brought by MSAs in patients with CRPC.

## Figures and Tables

**Table 1:** The therapeutics approved by the U.S. FDA for prostate cancer patients since 2004.

Category	Brand Name	Active Ingredient	Approved Date & Therapeutic Use
Chemotherapy	Taxotere	Docetaxel in combination with prednisone	19 May 2004 mCRPC
	Jevtana	Cabazitaxel in combination with prednisone	17 June 2010 mCRPC after docetaxel
	Zytiga	Abiraterone acetate in combination with prednisone	28 April 2011 mCRPC after docetaxel
	Zytiga	Abiraterone acetate in combination with prednisone	10 December 2012 mCPRC before chemotherapy
	Zytiga	Abiraterone acetate in combination with prednisone	7 February 2018 mCSPC
Hormonal therapy	Erleada	Apalutamide	14 February 2018 nmCRPC
	Erleada	Apalutamide	17 September 2019 nmCRPC
	XTANDI Capsules	Enzalutamide	31 August 2012 mCRPC after docetaxel
	XTANDI	Enzalutamide	13 July 2018 nmCRPC
	NUBEQA	darolutamide	30 July 2019 nmCRPC
	XTANDI	Enzalutamide	16 December 2019 CSPC
	Orgovyx	relugolix	18 December 2020 Advanced prostate cancer
Immunotherapy	Provenge	Sipuleucel-T	29 April 2010 Asymptomatic or minimally symptomatic mCRPC
Radiotherapy	Xofigo	Radium-223	15 May 2013 mCRPC
	Gallium 68 PSMA-11	Gallium 68 PSMA-11	1 December 2020 Detection and localization of prostate cancer
	Pylarify	Piflufolastat F18	26 May 2021 Identify prostate specific membrane lesions in prostate cancer
	Pluvicto	Lutetium (177 Lu) Vipivotide tetraxetan	23 March 2022 Prostate-specific membrane androgen-positive mCRPC

nmCRPC: non-metastatic castration-resistant prostate cancer. mCRPC: metastatic castration-resistant prostate cancer. mCSPC: metastatic castration-resistant prostate cancer.

**Table 2:** In vitro potency of MSAs as anti-prostate cancer agents.

Compound	IC_50_ Value	Cell Model	Bioassay Method	Reference
Paclitaxel	2.2 nM	DU145	Coulter counter	[23]
	4.0 nM	MDA PCa 2a	MTT	[24]
	6.2 nM	MDA PCa 2b	MTT	[24]
	1.6 nM	LNCaP	MTT	[24]
	10.3 nM	PC3	MTT	[24]
Epothilone A	13 nM	DU145	Coulter counter	[23]
	1.2 nM	MDA PCa 2a	MTT	[24]
	5.1 nM	MDA PCa 2b	MTT	[24]
	0.7 nM	LNCaP	MTT	[24]
	3.7 nM	PC3	MTT	[24]
Desoxyepothione A	200 nM	DU145	Coulter counter	[23]
Epothilone B	0.6 nM	DU145	Coulter counter	[23]
	0.7 nM	MDA PCa 2a	MTT	[24]
	1.0 nM	MDA PCa 2b	MTT	[24]
	0.2 nM	LNCaP	MTT	[24]
	0.2 nM	PC3	MTT	[24]
Desoxyepothilone B	1.7 nM	DU145	Coulter counter	[23]
26-Fluoroepothilone B	2.8 nM	MDA PCa 2a	MTT	[24]
	2.7 nM	MDA PCa 2b	MTT	[24]
	1.2 nM	LNCaP	MTT	[24]
	0.6 nM	PC3	MTT	[24]
protopine	13.0 µM	PC-3	SRB assay	[25]
	15.8 µM	DU145	SRB assay	[25]
(-)-zampanolide	2.9 nM	PC-3	Methylene blue	[26]
(-)-dactylolide	751 nM	PC-3	Methylene blue	[26]

## Data Availability

No new data were created.
